# Expression of methylation-related genes is associated with overall survival in patients with non-small cell lung cancer

**DOI:** 10.1038/sj.bjc.6604343

**Published:** 2008-04-15

**Authors:** J Xing, D J Stewart, J Gu, C Lu, M R Spitz, X Wu

**Affiliations:** 1 Department of Epidemiology, Unit 1340, The University of Texas MD Anderson Cancer Center, 1155 Pressler Boulevard, Houston, TX 77030, USA; 2 Department of Thoracic/Head & Neck Medical Oncology, The University of Texas MD Anderson Cancer Center, Houston, TX 77030, USA

**Keywords:** DNA methyltransferase, hypermethylation, non-small cell lung cancer, prognosis

## Abstract

The abnormality of DNA methylation is involved in tumour progression, and thus has a modulating effect on clinical outcome of cancer patients. In this study, we measured the mRNA expression levels of three methylation-regulating genes (*DNMT1*, *DNMT3b*, and *MBD2*) in 148 tumour samples from patients with non-small cell lung cancer (NSCLC) using quantitative real-time polymerase chain reaction and then determined their prognostic values. Our data showed that the high level of *DNMT1* expression was significantly associated with an increased risk of death in all NSCLC patients (hazard ratio (HR), 1.74; 95% confidence interval (95% CI), 1.04–2.90). However, the high level of *DNMT3b* expression was significantly associated with poor prognosis only in young patients (<65 years). The high level of *MBD2* expression had a significantly reduced risk for death only in male patients and in squamous cell lung carcinoma (SQLC) patients. All three combination groups with *DNMT1* and *DNMT3b*, *DNMT1* and *MBD2* or *DNMT3b* and *MBD2* revealed significant combined effects in male patients and SQLC patients. Our results suggest that DNMT1, DNMT3b, and MBD2 may play important roles in modulating NSCLC patient survival and thus be useful for identifying NSCLC patients who would benefit most from aggressive therapy.

The malignant transformation of human cells is usually driven by the activation of oncogenes or the inactivation of tumour suppressor genes, which can be caused by epigenetic events (Barrett *et al*, 1986; Simons, 1995; Jones and Laird, 1999). DNA methylation has been well documented as the major form of epigenetic gene regulation (Shames *et al*, 2007). Furthermore, an altered DNA methylation pattern is one of the most consistent epigenetic hallmarks of human cancer (Baylin, 2005; Esteller, 2005; Luczak and Jagodzinski, 2006). Usually, neoplastic cells simultaneously exhibit global genomic hypomethylation and region-specific hypermethylation. Most hypomethylation events in cancer cells appear to occur in repetitive and parasitic elements, which are heavily methylated in normal cells (Hoffmann and Schulz, 2005). These events may result in increased genomic instability. The hypermethylation of CpG islands in the promoter region of genes has been reported to cause the inactivation of tumour suppressor genes, which is critical to the process of tumorigenesis (Newell-Price *et al*, 2000; Teodoridis *et al*, 2004; McCabe and Caudill, 2005).

The exact nature of the defect in methylation machinery of tumour cells remain unclear; however, it may be related to the expression of DNA methyltransferases (DNMTs), primarily DNMT1, DNMT3a, and DNMT3b (Leonhardt and Bestor, 1993). DNMTs usually catalyse the transfer of methyl groups to CpG dinucleotides to produce either hemimethylated or fully methylated DNA strands. DNMT1 is best known as the maintenance methyltransferase that copies methylation patterns after DNA replication; DNMT3a and DNMT3b are primarily *de novo* methylators of CpG sites. Numerous studies (Issa *et al*, 1993; Robertson *et al*, 1999) have evaluated the expression of *DNMT*s in tumour tissue, and most have reported variable levels of overexpression, particularly of *DNMT1* and *DNMT3b*. Rhee *et al* (2002) demonstrated that DNMT1 and DNMT3b cooperatively maintain DNA methylation and gene silencing in human cancer cells. In contrast, DNA demethylase-methyl-CpG-binding domain 2 (MBD2) performs the reverse reaction to DNMTs (Bhattacharya *et al*, 1999). Compared to the adjacent normal tissue, a significant decrease in *MBD2* mRNA expression has been observed in various tumour tissue types (Kanai *et al*, 1999; Patra *et al*, 2002). These findings suggest that DNA methyltransferases and DNA demethylases play pivotal roles in the initiation and progression of tumours and thus may be useful in the clinical diagnosis and prognostic assessment of cancer.

Lung cancer is the leading cause of cancer-related death in the United States, with an estimated 160 390 deaths in 2007 (Jemal *et al*, 2007). The most common histological type is non-small cell lung cancer. The current 5-year survival rate of non-small cell lung cancer (NSCLC) is only 2–47% for different stages, and this has improved little over the past two decades. Thus, new prognostic markers are needed to help identify patients with poor prognoses, who may benefit from more aggressive treatment approaches. Previous studies have consistently reported that DNMT isoforms are significantly upregulated in human lung cancer cell lines and NSCLC tissue specimens (Sato *et al*, 2002; Vallbohmer *et al*, 2006). However, studies of the association between *DNMT* expression and clinical outcome in NSCLC patients have produced inconsistent results (Kim *et al*, 2006; Vallbohmer *et al*, 2006; Lin *et al*, 2007), and few have evaluated the prognostic value of *MBD2*. Therefore, we determined the expression of *DNMT1*, *DNMT3b*, and *MBD2* by real-time quantitative PCR in 148 tumour samples from NSCLC patients and analysed their prognostic value, both separately and jointly.

## MATERIALS AND METHODS

### Patients and tissue specimens

One hundred forty-eight patients with histologically confirmed NSCLC were included in this study, all of whom were recruited from 1993 to 1997 and had undergone curative surgical resections at The University of Texas MD Anderson Cancer Center. There were no age, sex, ethnicity, or tumour stage restrictions on patient enrolment. Fresh tumour tissues were frozen immediately after excision and stored in liquid nitrogen until they were used for RNA extraction. Demographic and clinical data were collected from the patient history database and chart review at MD Anderson Cancer Center. The survival duration was evaluated as of June 2005. Study approval was obtained from the MD Anderson institutional review board.

### RNA extraction and cDNA synthesis

Total RNA extraction and cDNA synthesis were performed as previously described (Lin *et al*, 2006). In brief, frozen tissues were homogenised, and total RNA was isolated with the EZNA total RNA kit (Omega Bio-tek, Doraville, GA, USA) according to the manufacturer's instructions. RNA was eluted in RNase-free water, and the concentration was determined by spectrophotometer (Beckman Coulter, Fullerton, CA, USA). The quality of RNA samples was determined by 1% agarose gel electrophoresis and ethidium bromide-staining. The cDNA synthesis reaction was then carried out using the Taqman reverse transcription reagents kit (Applied Biosystems, Branchburg, NJ, USA) in a final volume of 20 *μ*l containing 1 × RT buffer, 5 mmol l^−1^ MgCl_2_, 250 *μ*mol l^−1^ each dNTP, 20 units of RNase inhibitor, 50 units of multiscribe reverse transcriptase, 2.5 *μ*mol l^−1^ random hexamers, and 0.5 *μ*g of total RNA. The reaction mixtures were incubated at ambient temperature for 10 min and then at 42°C for 30 min. Reverse transcriptase was inactivated by heating at 99°C for 5 min. All cDNA products were stored at −30°C until they were used for the real-time PCR.

### Real-time PCR

The primers and probes for real-time PCR were designed using Primer Express software (version 2.0, Applied Biosystems). We confirmed the specificity of primers and probes and the absence of single nucleotide polymorphisms by searching the Genebank database. To avoid amplifying residual genomic DNA, one of the two primers or the probe was designed across the junction region between two exons. The sequences of the primers and probes were as follows: *DNMT1*, forward primer (FP): 5-AGAAGAGACGTAGAGTTACATCCAGAGA-3, reverse primer (RP): 5-GCGTTCCTGATTTTGCTCTTTC-3, probe: 5-FAM- CGAGTTGCTAGACCGCTTCCTGC AGA-TAMRA-3; *DNMT3b*, FP: 5-CGCACCCCGGAGATCA-3, RP: 5-ACTGGACACCTCCC TCTTGGA-3, probe: 5-FAM-AGGCCGAAGATCAAGCTCGCGACT-TAMRA-3; *MBD2*, FP: 5-AGTGAAATCAGACCCACAACGA-3, RP: 5-GCACTAAGTCCT TGTAGCCTCTTCTC-3, probe: 5-FAM-TGAATGAACAGCCACGTCAGCTTTTCTATAMRA-3; and *GAPDH*, FP: 5-AAGGCTGAGAACGGGAAGC-3, RP: 5-GAGGGATCTCGC TCCTGGA-3, probe: 5-FAM-TGTCATCAATGGAAATCCCATCACCATC-TAMRA-3.

PCR amplification and real-time detection of product were performed using the ABI Prism 7900 sequence detection system (Applied Biosystems) in a 10-*μ*l reaction mixture consisting of 1 × Taqman buffer A, 3.4 mM MgCl_2_, 100 *μ*M each dNTP, 0.2 *μ*M each primer, 0.1 *μ*M probe, 0.02 U of AmpliTaq Gold DNA polymerase, and 1 *μ*l of each synthesized cDNA template. The thermal cycling conditions comprised one cycle at 95°C for 10 min and 40 cycles at 95°C for 15 s, 60°C for 1 min. A relative quantification method using standard curve was used to measure the relative expression levels of each gene in all samples. In brief, the cDNA product of commercial human total RNA (Stratagene, La Jolla, CA, USA) was serially diluted by fivefold per dilution to produce a 6-point standard curve for each tested gene. The quantity of tested gene in each sample was arbitrarily measured as the dilution level of standard sample. The human *GAPDH* gene was used as an internal control to normalise the RNA input amount, reverse transcription efficiency, and RNA quality. The relative expression levels of each sample were expressed as N-fold expression differences in the target gene relative to the *GAPDH* genes. The PCR reaction for each sample was duplicated, and the mean value was used in the statistical analysis.

### Statistical analysis

We used STATA statistical software, version 8.0 (Stata Corp., College Station, TX, USA), for all statistical analyses. Smoking status and pack-years were categorised as previously described (Wu *et al*, 2003). Overall survival duration was defined as the time from lung cancer diagnosis to the date of patient death or last follow-up. Survival status was evaluated using Pearson's *χ*^2^ test or Fisher's exact test for categorical variables and Student's *t*-test for continuous variables. The expression levels of all three genes were dichotomised as high or low, with the median values of normalised mRNA expression used as the cutoff points. The hazard ratios and 95% confidence intervals were calculated using Cox proportional hazards regression analysis to determine the effects of gene expression level on overall survival. Multivariate analysis was used to control potential confounding factors (age, sex, ethnicity, smoking status, tumour grade, and clinical disease stage). Kaplan–Meier plots and the log-rank test were used to evaluate the association between survival duration and the expression of the three genes. Because only three genes were involved in our study, multiple testing issues have not been considered for our analysis. All reported *P*-values were based on two-sided tests, and the level of significance was set at *P*<0.05.

## RESULTS

### Distribution of demographic and clinical variables

Patients' demographic and clinical characteristics are summarised in Table 1. One hundred forty-eight NSCLC patients were included in this study, with a mean age of 65.0 years. Ninety-four percent of patients were white, and 92% were ever smokers. The most common histologic tumour types were lung adenocarcinoma (47%) and SQLC (36%). Forty-eight percent of patients were at stage I, 15% at stage II, 23% at stage III and 14% unknown. However, tumours were mostly at intermediate (grade 2 (38.5%)) or low (grade 3 (39%)) differentiations. The median survival duration was 45.9 months, and the 5-year overall survival rate was 42%. Among 136 patients with complete treatment information, 77 (57%) patients received surgery alone and 59 (43%) patients received surgery with adjuvant chemo or radiotherapy. As shown in Table 1, there were no significant differences in the age (*P*=0.16), sex (*P*=0.22), ethnicity (*P*=0.54), smoking status (*P*=0.42), pack-years (*P*=0.66), or histologic tumour subtype (*P*=0.79) between patients who were alive and dead at last follow-up. Nevertheless, the high stage at diagnosis was a significant risk predictor for death (*P*<0.001) in all patients, followed by high grade (*P*=0.09). In addition, when we compare patients with surgery alone, patients with adjuvant therapy had a significantly higher death rate (*P*<0.001), mainly because adjuvant therapy, as a secondary treatment, was mostly applied to patients of high stages and at high risks of recurrence and metastasis.

### Association between DNMT1, DNMT3b, and MBD2 expression and overall survival

**Figure 1:**
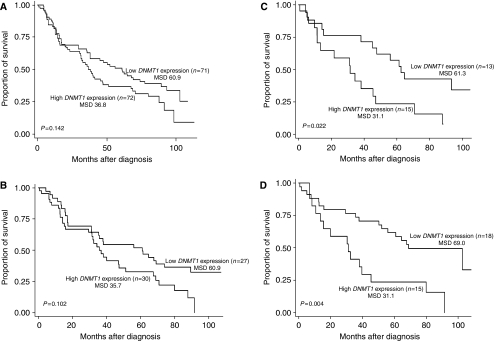
Kaplan–Meier survival estimates for patients with NSCLC. Survival duration by *DNMT1* expression (**A**) in all patients, (**B**) in male patients, (**C**) in patients who were heavy smokers and (**D**) in SQLC patients; MSD, Median survival duration.

The median expression values for *DNMT1, DNMT3b*, and *MBD2* genes are 1.363, 39.425 and 2.978, respectively. No significant correlation was observed between the mRNA expression of *DNMT1*, *DNMT3b*, *and MBD2* and the tumour grade, stage, and histological type (data not shown). We dichotomised the expression level of these three genes into high and low groups by using the median value as the cutoff. According to the results of the multivariate Cox proportional hazards model, DNMT1 expression was significantly associated with patient survival rate of NSCLS patients. After adjusting for age, sex, ethnicity, smoking status, tumour grade, and clinical disease stage, we found that the high level of *DNMT1* expression was associated with a 74% increased risk of death (HR, 1.74; 95% CI, 1.04–2.90). However, no statistically significant association was found for *DNMT3b* (HR, 1.27; 95% CI, 0.78–2.06) and *MBD2* (HR, 0.80; 95% CI, 0.48–1.35) (Table 2). The Kaplan–Meier analysis revealed that patients with the high level of *DNMT1* expression had notably shorter median survival duration (36.8 months) than did those with the low level of expression (60.9 months) although it was not statistically significant (*P*=0.142, log-rank test) (Figure 1A). Moreover, we found no significant difference between the median survival durations of NSCLC patients with low and high levels of *DNMT3b* or *MBD2* expression (data not shown).

In stratified analyses (Table 2), our data showed that the high level of *DNMT1* expression was associated with a high risk of death in male (HR, 2.17; 95% CI, 1.11–4.28) but not in females (HR, 1.63; 95% CI, 0.59–4.49), in heavy smokers (HR, 5.67; 95% CI, 1.64–19.60) but not in light smokers (HR, 1.08; 95% CI, 0.54–2.19), and in SQLC patients (HR, 3.26; 95% CI, 1.22–8.70) but not in adenocarcinoma patients (HR, 1.47; 95% CI, 0.64–3.37). The Kaplan–Meier analyses (Figure 1B–D) also showed that, compared with the low level of *DNMT1* expression, the high level of expression was only notably associated with short median survival duration in males (35.7 *vs* 60.9 months, *P*=0.102), in heavy smokers (31.1 *vs* 61.3 months, *P*=0.022), and in SQLC patients (31.1 *vs* 69.0 months, *P*=0.004). Furthermore, a high level of *DNMT3b* expression was significantly associated with poor prognosis in patients aged <65 years (HR, 2.76; 95% CI, 1.12–6.76). In comparison, a high level of *MBD2* expression provided significant protective effect in male patients (HR, 0.40; 95% CI, 0.17–0.94) and SQLC patients (HR, 0.35; 95% CI, 0.12–1.03). The Kaplan–Meier analyses for *DNMT3b* and *MBD2* expressions indicated the similar results (data not shown). In addition, we performed stratified analysis of the three genes by adjuvant therapy (Table 2). We didn't find any notable effects of adjuvant therapy on the prognostic values of three genes expression.

### Combined analysis on the prognostic value of DNMT1, DNMT3b, and MBD2 in different subgroups

On the basis of our initial findings, we defined high expression of *DNMT1* and *DNMT3b* and low expression of *MBD2* as three unfavourable factors that were associated with poor survival. We determined the combined effect of either two genes on overall survival in different subgroups of patients stratified by age, sex and histological type (Table 3). All three combination groups with *DNMT1* and *DNMT3b*, *DNMT1* and *MBD2* or *DNMT3b* and *MBD2* revealed significant combined effects in male patients (*P* for trend=0.023, <0.001 and =0.010, respectively) and SQLC patients (*P* for trend=0.022, 0.003 and 0.019, respectively), but not in female patients (*P* for trend=0.357, 0.243 and 0.341, respectively) and adenocarcinoma patients (*P* for trend=0.245, 0.487 and 0.431, respectively). Findings from Kaplan–Meier analyses for all three combination groups also indicated that the median overall survival duration was substantially declined as the number of unfavourable factors increased in male patients and SQLC patients but not in female patients and adenocarcinoma patients (data not shown). In comparison, we found that age exerted different effects on the prognostic value of three combination groups.

## DISCUSSION

In this study, we measured the mRNA expression level of three methylation-related genes (*DNMT1*, *DNMT3b* and *MBD2)* in NSCLC tumour tissues using quantitative real-time PCR and evaluated their prognostic values. Our data showed that these three genes had modulating effects on clinical outcome of NSCLC patients.

We found that the high level of *DNMT1* expression was significantly associated with poor overall survival in NSCLC patients, independent of tumour stage and grade. Kim *et al* (2006) reported similar results in a study of 102 NSCLC patients (HR 3.51; 95% CI, 1.18–12.76). Lin *et al* (2007) further verified the association between the high level of *DNMT1* expression and poor prognosis of lung cancer at protein level. In a mouse model of prostate cancer, McCabe *et al* (2006) also found an association between *DNMT1* and survival and demonstrated that treatment with 5-aza, a DNA methyltransferase inhibitor, prevented the development of lymph node metastases and dramatically improved survival. However, in a study of 91 NSCLC patients, no association was found between *DNMT1* mRNA expression and clinical outcome (Vallbohmer *et al*, 2006). This discrepancy may be a result of different patient populations and different methods for normalisation of mRNA expression.

The molecular mechanism of DNMT1 survival modulation remains to be elucidated. Several previous studies (Paz *et al*, 2003; Robert *et al*, 2003) have suggested that *DNMT1*, as a major methylation-inducing factor, is needed to maintain CpG methylation and aberrant gene silencing in human cancer cells. Kim *et al* (2006) found that elevated mRNA levels of *DNMT1* were significantly associated with promoter hypermethylation of tumour suppressor gene p16 in NSCLC patients. Lin *et al* (2007) demonstrated that DNMT1 overexpression was associated with the hypermethylation of *FHIT*, *p16*^*ink4a*^, and *RARβ* and that the promoters of methylated *FHIT*, *p16*^*ink4a*^, and *RARβ* were bound by DNMT1 protein. These findings suggest that *DNMT1* hypermethylates survival-associated tumour suppressor genes, leading to their functional inactivation. However, a recent report (Sato *et al*, 2002) found no significant association between *DNMT1* expression and DNA methylation of some tumour-associated genes, suggesting that DNMT1 also contributes to cancer development and progression through alternative pathways. For example, Chuang *et al* (1997) reported that DNMT1 is in a complex with proliferating cell nuclear antigen, a factor that assists in DNA replication, suggesting that DNMT1 may play a vital role in replication except for regulating gene expression. Egger *et al* (2006) also demonstrated that DNMT1 is essential to the proliferation and survival of cancer cells. In addition, Biniszkiewicz *et al* (2002) reported that the overexpression of DNMT1 resulted in the activation of silent alleles in Igf2 gene by *de novo* methylation, thus leading to increased cell proliferation and overgrowth.

We found no significant association between *DNMT3b* expression and survival in all NSCLC patients. This result is consistent with those of Kim *et al* (2006) and Vallbohmer *et al* (2006). However, possibly because of a limited number of samples, neither of these studies included an analysis stratified by patient characteristics. Our stratified data analysis revealed a significant association between the high level of *DNMT3b* expression and increased risk of death in patients aged <65 years, suggesting that *DNMT3b* expression may be an age-related prognostic predictor. Similarly, Girault *et al* (2003) found that *DNMT3b* overexpression was associated with a short relapse-free survival duration in a subgroup of breast cancer patients, and Wang *et al* (2004) demonstrated that increased *DNMT3b* promoter activity (resulting from a C-to-T polymorphism) led to lower survival duration in patients with SQLC of the head and neck. The functional mechanism of DNMT3b in prognosis is still unclear. Unlike *DNMT1*, *DNMT3b* has been suggested to be site-selective in the regulation of aberrant gene silencing. *DNMT3b* expression has also been found to be essential for cancer cell survival by inhibiting apoptosis of tumour cells but not normal cells (Beaulieu *et al*, 2002). The results of *in vitro* studies by Geiman *et al* (2004) further indicate that *DNMT3b* contributes to gene silencing by recruiting chromatin remodeling histone deacetylase.

In this study, we also found that the high level of *MBD2* expression was associated with a significant protective effect in male patients (HR, 0.40; 95% CI, 0.17–0.94) and SQLC patients (HR, 0.35; 95% CI, 0.12–1.03). MBD2 has been found to catalyse demethylation by directly removing methyl groups from 5-methylcytosine residues in DNA (Bhattacharya *et al*, 1999). The results of our previous study (Zhu *et al*, 2004) suggest that *MBD2* expression prevents age-related, sex-related, and smoking-induced hypermethylation. Several studies have demonstrated altered *MBD2* mRNA expression in various tumour tissue specimens, but the findings have been inconsistent. A significant reduction in the level of *MBD2* mRNA expression was found in human colorectal and gastric tissues compared with nonmalignant tissues (Kanai *et al*, 1999), whereas elevated expression was reported in breast cancer (Billard *et al*, 2002). These differences may be explained by MBD2's dual functions (transcription repression and demethylation). To date, the specific role of MBD2 in NSCLC remains to be determined. In the present study, NSCLC tumour tissues had significantly lower levels of *MBD2* expression than did adjacent normal tissues (data not shown), suggesting that the effect of *MBD2* expression on clinical outcome may be related to demethylation. However, more conclusive evidence is needed.

Methylation varies as a function of age, sex, and smoking status (Ahuja and Issa, 2000; Toyooka *et al*, 2003). Therefore, it is plausible for our study to indicate that age, gender and smoking may modify the prognostic value of methylation-related genes in NSCLC patients. In addition, histologic tumour type-related differences in methylation have been documented in many genes, particularly tumour suppressor genes (Kim *et al*, 2004). Accordingly, our data revealed a histology-related prognostic significance of *DNMT1* and *MBD2* expression in NSCLC patients. To comprehensively evaluate the prognostic value of *DNMT1*, *DNMT3b*, and *MBD2*, we performed a combined analysis. Our results suggest that these genes may be useful for determining prognosis in NSCLC patients, particularly in male patients and SQLC patients. Nevertheless, the results of our stratified and combined analyses should be interpreted cautiously because of the small sample size in each stratum and empirical cutoff point chooses. A larger study is needed to verify these findings.

To conclude, our findings further clarify the inconsistent findings of previous reports. We found that elevated *DNMT1* mRNA expression was significantly associated with poor prognosis in NSCLC patients. *DNMT3b* and *MBD2* had potential age-, sex-, and histology-related prognostic value in NSCLC patients, respectively. In addition, there is a significant combined effect of these three genes on overall survival. Therefore, these three genes could be useful in predicting clinical outcome in NSCLC patients and thus identifying patients who would benefit from aggressive therapy.

## Figures and Tables

**Table 1:** Distribution of demographic and clinical variables by survival status

	**Alive**		**Death**		
	**(*n*=49)**		**(*n*=99)**		
**Variable**	**Number**	**(%)**	**Number**	**(%)**	***P*-value**
*Gender*					
Male	24	(49)	59	(60)	
Female	25	(51)	40	(40)	0.22
					
*Ethnicity*					
Caucasian	45	(92)	94	(95)	
Mexican American	1	(2)	2	(2)	
African American	2	(4)	3	(3)	
Others	1	(2)	0	(0)	0.54
					
*Smoking status* a					
Never smoker	6	(13)	6	(6)	
Former smoker	17	(38)	36	(40)	
Current smoker	22	(49)	49	(54)	0.42
					
*Histology*					
Adenocarcinoma	23	(47)	46	(46)	
SQLC	19	(39)	34	(34)	
Other	7	(14)	19	(20)	0.79
					
*Tumour stage* a					
I	33	(79)	38	(45)	
II	5	(12)	17	(20)	
III	4	(10)	30	(35)	<0.001
					
*Grade* a					
I	7	(18)	5	(6)	
II	16	(41)	41	(46)	
III	16	(41)	42	(48)	0.09
					
*Treatment*					
Surgery alone	35	(45)	42	(55)	
Surgery with adjuvant therapy	9	(15)	50	(85)	<0.001
					
Age in years (mean±s.d.)	63.3±10.5		65.9±10.8		0.16
Pack-years^b^ (mean±s.d.)	50.1±33.0		52.6±24.7		0.66

a Few patients did not have documented smoking status, stage or grade information at the time of this investigation.

b For ever smokers only

**Table 2:** Association of *DNMT1*, *DNMT3b* and *MBD2* mRNA expression in tumour tissues with NSCLC patient survival

	***DNMT1* a**			***DNMT3b* a**			***MBD2* a**		
**MRNA expression^b^**	**Alive**	**Dead**	**HR^c^ (95% CI)**	**Alive**	**Dead**	**HR^c^ (95% CI)**	**Alive**	**Dead**	**HR^c^(95% CI)**
*Overall*									
Low	26	45	ref.	25	46	ref.	21	50	ref.
High	20	52	1.74 (1.04–2.90)	21	51	1.27 (0.78–2.06)	25	47	0.80 (0.48–1.35)
									
*Age* d									
<65 years									
Low	12	18	ref.	15	17	ref.	10	24	ref.
High	9	24	3.08 (1.26–7.51)	6	25	2.76 (1.12–6.76)	11	18	1.40 (0.46–4.23)
⩾65 years									
Low	13	27	ref.	9	29	ref.	10	26	ref.
High	10	28	2.23 (1.09–4.55)	14	26	0.74 (0.37–1.48)	13	29	0.66 (0.33–1.30)
									
*Sex*									
Male									
Low	16	27	ref.	14	27	ref.	10	30	ref.
High	6	30	2.17 (1.11–4.28)	8	30	1.63 (0.82–3.24)	12	27	0.40 (0.17–0.94)
Female									
Low	10	18	ref.	11	19	ref.	11	20	ref.
High	14	22	1.63 (0.59–4.49)	13	21	1.29 (0.53–3.15)	13	20	0.86 (0.34–2.14)
									
*Pack-years* e									
<52									
Low	14	26	ref.	10	20	ref.	10	25	ref.
High	9	23	1.08 (0.54–2.19)	13	29	1.02 (0.51–2.03)	13	24	0.72 (0.34–1.50)
⩾52									
Low	8	13	ref.	9	15	ref.	7	19	ref.
High	2	15	5.67 (1.64–19.6)	1	13	1.60 (0.57–4.47)	3	9	0.66 (0.14–3.15)
									
*Treatment* e									
Surgery alone									
Low	16	22	ref.	18	26	ref.	14	21	ref.
High	16	19	1.64 (0.71–3.77)	414	159	0.73 (0.31–1.70)	18	20	0.72 (0.29–1.77)
Surgery and adjuvant therapy									
Low	7	21	ref.	4	18	ref.	5	26	ref.
High	2	29	2.16 (0.97–4.84)	5	32	1.22 (0.63–2.38)	4	24	0.84 (0.37–1.93)
									
*Histology*									
Adenocarcinoma									
Low	9	16	ref.	11	21	ref.	8	22	ref.
High	13	29	1.47 (0.64–3.37)	11	24	1.48 (0.72–3.04)	14	23	1.21 (0.43–3.43)
SQLC									
Low	16	18	ref.	12	15	ref.	11	19	ref.
High	2	15	3.26 (1.22–8.70)	6	18	1.56 (0.58–4.24)	7	14	0.35 (0.12–1.03)

a Number of patients may not add up to the total number due to assay failure.

b Dichotomised using the median value for mRNA expression in tumour tissues. The cutoff points for *DNMT1*, *DNMT3b*, and *MBD2* were 1.362, 39.425 and 2.978, respectively.

c Adjusted for age, sex, ethnicity, smoking status, tumour grade and clinical disease stage when appropriate.

d Dichotomised using the median value in all patients.

e Dichotomised using the median value in ever smokers.

**Table 3:** Combined effect of DNMT1and DNMT3b, DNMT1 and MBD2 or DNMT3b and MBD2 on survival stratified by age, sex, and histology

	***DNMT1* and *DNMT3b*^a^**			***DNMT1* and *MBD2*^a^**			***DNMT3b* and *MBD2*^a^**		
**No. of unfavourable event^b^**	**Alive (%)**	**Death (%)**	**Adjusted HR^c^ (95% CI)**	**Alive (%)**	**Death (%)**	**Adjusted HR^c^ (95% CI)**	**Alive (%)**	**Death (%)**	**Adjusted HR^c^ (95% CI)**
*Age<65* d									
0	11 (52)	10 (24)	Ref.	3 (14)	5 (12)	Ref.	7 (33)	6 (14)	Ref.
1	5 (24)	15 (36)	2.11 (0.62–7.13)	17 (81)	26 (62)	0.34 (0.07–1.62)	12 (57)	23 (55)	2.50 (0.61–10.21)
2	5 (24)	17 (40)	4.96 (1.68–14.6)	1 (5)	11 (26)	2.26 (0.43–11.79)	2 (10)	13 (31)	2.89 (0.65–12.88)
			*P* for trend=0.003			*P* for trend=0.037			*P* for trend=0.193
									
*Age⩾65* d									
0	6 (26)	16 (29)	Ref.	5 (22)	11 (20)	Ref.	3 (13)	8 (15)	Ref.
1	10 (44)	24 (44)	1.31 (0.54–3.18)	16 (70)	34 (64)	1.84 (0.76–4.43)	16 (70)	42 (76)	1.45 (0.51–4.16)
2	7 (30)	15 (27)	1.50 (0.60–3.78)	2 (8)	10 (16)	5.82 (1.76–19.28)	4 (17)	5 (9)	1.33 (0.30–5.91)
			*P* for trend=0.395			*P* for trend=0.007			*P* for trend=0.683
									
*Male*									
0	12 (55)	17 (30)	Ref.	7 (31)	9 (16)	Ref.	6 (27)	7 (12)	Ref.
1	6 (27)	20 (35)	1.66 (0.72–3.82)	14 (64)	36 (64)	3.29 (1.11–9.73)	14 (64)	40 (70)	3.74 (1.33–10.52)
2	4 (18)	20 (35)	2.71 (1.15–6.41)	1 (5)	12 (21)	10.7 (2.86–39.8)	2 (9)	10 (18)	4.91 (1.43–16.89)
			*P* for trend=0.023			*P* for trend<0.001			*P* for trend=0.010
									
*Female*									
0	6 (25)	9 (22)	Ref.	1 (4)	7 (18)	Ref.	4 (17)	7 (18)	Ref.
1	9 (38)	19 (48)	1.49 (0.53–4.19)	21 (88)	24 (60)	0.80 (0.24–2.64)	16 (66)	25 (62)	1.11 (0.23–5.35)
2	9 (38)	12 (30)	1.77 (0.53–5.89)	2 (8)	9 (22)	4.60 (0.64–32.8)	4 (17)	8 (20)	2.05 (0.29–14.5)
			*P* for trend=0.357			*P* for trend=0.243			*P* for trend=0.341
									
*SQLC*									
0	11 (61)	9 (27)	Ref.	5 (28)	7 (21)	Ref.	4 (22)	3 (9)	Ref.
1	6 (33)	15 (46)	1.91 (0.66–5.51)	13 (72)	18 (55)	2.89 (0.75–11.1)	11 (61)	23 (70)	4.59 (0.55–38.03)
2	1 (6)	9 (27)	7.80 (1.58–38.46)	0	8 (24)	9.64 (2.20–42.3)	3 (17)	7 (21)	10.95 (1.13–105.78)
			*P* for trend=0.022			*P* for trend=0.003			*P* for trend=0.019
									
*Adenocarcinoma*									
0	6 (28)	9 (20)	Ref.	3 (14)	6 (13)	Ref.	5 (23)	9 (20)	Ref.
1	8 (36)	19 (42)	2.50 (0.93–6.70)	17 (77)	27 (60)	0.33 (0.08–1.45)	15 (68)	26 (58)	1.23 (0.43–3.53)
2	8 (36)	17 (38)	1.94 (0.71–5.28)	2 (9)	12 (27)	0.87 (0.17–4.49)	2 (9)	10 (22)	1.74 (0.43–7.06)
			*P* for trend=0.245			*P* for trend=0.487			*P* for trend=0.431

a Number of patients may not add up to the total number due to assay failure.

b Unfavourable factors were defined as high mRNA expression for *DNMT1* and *DNMT3b* or low mRNA expression for *MBD2*.

c Adjusted for age, sex, ethnicity, smoking status, tumour grade and clinical disease stage when appropriate.

d Dichotomised using the median value in all patients.
